# The Relationship between Mechanical Hyperalgesia Assessed by Manual Tender Point Examination and Disease Severity in Patients with Chronic Widespread Pain: A Cross-Sectional Study

**DOI:** 10.1155/2014/417596

**Published:** 2014-04-15

**Authors:** Kirstine Amris, Eva Ejlersen Wæhrens, Anders Jespersen, Anders Stockmarr, Robert Bennett, Henning Bliddal, Bente Danneskiold-Samsøe

**Affiliations:** ^1^The Parker Institute, Department of Rheumatology, Copenhagen University Hospital Bispebjerg and Frederiksberg Hospital, Nordre Fasanvej 57, 2000 Frederiksberg, Denmark; ^2^Institute of Public Health, University of Southern Denmark, 5000 Odense, Denmark; ^3^Section for Statistics and Data Analysis, Department of Applied Mathematics and Computer Science, Technical University of Denmark, 2800 Lyngby, Denmark; ^4^Oregon Health & Science University, Portland, OR 97239, USA

## Abstract

The clinical utility of tender point (TP) examination in patients reporting chronic widespread pain (CWP) is the subject of contemporary debate. The objective of this study was to assess the relationship between mechanical hyperalgesia assessed by manual TP examination and clinical disease severity. 271 women with CWP were recruited from a clinical setting. Data collection included patient-reported symptoms, health-related quality of life variables, and observation-based measures of functional ability, muscle strength, 6-minute walk, and pressure pain thresholds measured by cuff algometry. TP examination was conducted according to ACR-guidelines. Relationships between disease variables and TP count (TPC) were analyzed with logistic regression in a continuum model, allowing the TPC to depend on the included disease variables and two regression models carried out for a TPC threshold level, varying between 1 and 17. The threshold analyses indicated a TPC threshold at 8, above which a large number of disease variables became consistently significant explanatory factors, whereas none of the disease variables reached a significance level in the continuum model. These results support the premise that the presence of mechanical hyperalgesia influences symptomatology in CWP and that the severity of clinical expression is related to a threshold of TPs, rather than being part of a continuum.

## 1. Introduction 

Fibromyalgia is a characterized subgroup of patients presenting with chronic widespread pain (CWP) and widespread mechanical hyperalgesia. These characteristics are enshrined in the 1990-ACR criteria [1] that have been the cornerstone for research studies of the past 2 decades. Contemporary research in subjects fulfilling 1990-ACR criteria has provided persuasive evidence for augmented central pain processing in terms of sensitization of nociceptive neurons and ascending spinal tracts accompanied by dysfunction of descending pain inhibitory pathways [2]. The underlying pain mechanisms in subjects with CWP with fewer than 11 tender points (TP) are less well described [2]. Mechanical hyperalgesia is a clinical manifestation of central sensitization and, although an imperfect measure, the manual TP examination has been considered a primary identifier of pain hypersensitivity [3–6]. In the development of the 1990 ACR classification criteria for fibromyalgia, TPs were found to be the most powerful discriminator between fibromyalgia and control subjects; the best separation occurred at about the 13 TPs for mild tenderness (the subject state that palpation is painful) and about 6 TPs for moderate or greater tenderness (the pain complaint is accompanied by facial expression and/or flinch at palpation) [1]. In the clinical context, the 1990-ACR criteria cutoff at 11 TPs, based on a score of mild or greater tenderness, has been criticized for placing a diagnosis of fibromyalgia at the far end of a severity spectrum and for ignoring other key symptoms [7–9]. This has led to the suggestion of diagnostic criteria based on pain and typical fibromyalgia symptoms, but omitting the evaluation of mechanical hyperalgesia [9]. Influential in the development of these new symptom-based diagnostic criteria is the argument that fibromyalgia is best understood as part of a polysymptomatic distress continuum and not as a categorical disorder. A contrary opinion is that fibromyalgia is both a categorical disorder and the end of a continuum of pain processing [10].

A positive relationship between TP count (TPC) and psychological distress seems well established, although a quite variable association between the TPC and clinical disease severity has been reported [11–15]. In these studies clinical disease severity has been defined in different ways and only a minority has included patients with CWP and fewer than 11 TPs or disease variables derived from observation-based assessment.

The WHO international classification of functioning, disability, and health (ICF) can serve as a framework when defining clinical disease severity [16]. The ICF conceptualize health and health-related states as a complex interplay of the following health components: body functions and structures (including mental functions), activities (execution of tasks) and participation (involvement in life situations), and environmental and personal factors. Based on the ICF, international recommendations for a number of musculoskeletal pain disorders have been developed [17], including definition and validation of ICF core sets for patients with CWP [18–23]. ICF core sets represent a selection of ICF categories relevant for specific conditions and serve as standards for the multidimensional assessment of patients for clinical encounters and trials.

The objective of this study was to evaluate the relationship between TPC threshold and clinical disease severity in patients with CWP using the multidimensional ICF measurement framework. It was hypothesized that the clinical disease burden in patients with CWP would be influenced by the presence and spread of mechanical hyperalgesia, as evaluated by the manual TP examination.

## 2. Methods

### 2.1. Study Design and Setting

The study was cross-sectional with systematic data collection on consecutive female patients diagnosed with CWP at rheumatologic examination and accepted for enrolment in an interdisciplinary rehabilitation program tailored for this patient population at the department of rheumatology, Frederiksberg Hospital. A comprehensive baseline assessment based on the brief ICF core set for CWP [18] was implemented. Several self-report and observation-based assessment tools were applied in the data collection and data stored in a clinical database. Data were obtained in a two-year period from 1 March, 2007, to 28 February, 2009. All examination methods were approved by the local ethics committee (KF 01-045/03).

### 2.2. Participants

The referral diagnosis of CWP was based on the 1990-ACR definition of wide spread pain (i.e., patient reporting of pain axially and in minimum 3 body quadrants) [1] and the diagnostic assessment prior to referral included a full rheumatologic examination and extensive blood test screening. Exclusion criteria were need of assistance in personal activities of daily living, concurrent history of major psychiatric disorder not related to the pain disorder, and other medical conditions capable of causing patients symptoms (e.g., uncontrolled inflammatory/autoimmune disorder, uncontrolled endocrine disorder, malignancy, etc.). All patients continued usual medication including analgesics.

### 2.3. Data Sources and Measurements

The brief ICF core set for CWP and applied instruments classified according to the dimensions of the ICF model are presented in Table 1.

#### 2.3.1. Patient-Reported Outcomes (PRO)

PRO were based on validated self-administered questionnaires. For some of the applied instruments content comparison and linkage with ICF categories have been reported in the literature [22]. Detailed descriptions are enclosed in Annex A (see Supplementary Material available online at http://dx.doi.org/10.1155/2014/417596). Additional data regarding health and personal and environmental factors were collected using a standardized basic information questionnaire (BIQ), for example, employment status, use of social security and health care services, pain medication, and family relationships.

#### 2.3.2. Observation-Based Outcomes

*Manual TP Examination and TPC.* TPs were assessed both for number and severity of mechanical hyperalgesia as described in the 1990-ACR guidelines [1]. Digital pressure of approximately 4 kg was applied at each of the 18 predefined TP sites, and the patient's pain response at each site was scored as 0 = no pain, 1 = mild pain (complaint of pain without grimace, flinch, or withdrawal), 2 = moderate pain (pain plus grimace or flinch), and 3 = severe pain (pain plus marked flinch or withdrawal). As the primary goal of this study was to evaluate the relationships between mechanical hyperalgesia and disease severity, a mild pain response (i.e., 1) was considered potentially ambiguous and the cutoff response was therefore set at ≥2. All TP examinations were performed by experienced and calibrated raters.

*Assessment of Motor and Process Skills (AMPS).* The AMPS is a standardized observation-based assessment instrument that incorporates the use of Rasch analysis providing equal-interval linear measures of the quality of ADL task performances [24, 25]. Two domains of ADL performance are evaluated: ADL motor skills (moving self and objects) and ADL process skills (organizing and adapting actions). Several studies support good test-retest and rater reliability as well as validity of the AMPS including in CWP populations [26]. AMPS evaluations were performed by trained and calibrated AMPS raters.

*Assessment of Pressure Pain Threshold and Tolerance.* Pressure pain sensitivity was determined on the lower leg using computerized cuff pressure algometry (CPA). The setup consisted of a pneumatic tourniquet cuff, a computerized compressor, and an electronic 10 cm visual analogue scale (VAS). Double-chambered textile tourniquet cuffs (VBM Medizintechnik GmbH, Sulz, Germany) were used for pressure application [27]. The following parameters were determined:* pain threshold *defined as the pressure of the cuff at the subject's first sensation of pain when applying a constantly raising pressure (Unit kPa);* pain tolerance *defined as the pressure of the cuff when the pressure is switched off by the patient due to worst tolerable pain caused by pressure stimulation (Unit kPa). Reduced pressure-pain thresholds assessed by CPA have been demonstrated in patients with fibromyalgia and reported not to be influenced by psychological distress, indicating that this method may be used for objective assessment of deep tissue pain hypersensitivity [28].

Baseline assessment also included measurements of maximal isokinetic knee muscle strength, maximal grip strength, and a 6-minute walk test (see Annex A).

### 2.4. Statistical Methods and Threshold Definition

Disease variables classified according to the ICF are presented as mean, standard deviation (SD), range, and number of people in the study population. Differences between groups were assessed using analysis of variance (*z*-test), with significance level 0.05.

Three different statistical models were applied in the multivariate analyses. First, TPC were analyzed with a logistic regression model, allowing the TPC to depend on the included disease variables from the ICF-measurement framework (the continuum model).

Secondly, each possible value for the TPC except the biggest possible (i.e., 1–17) was considered a threshold, with possibly different disease severity for patients with TPC above and below the threshold. For each threshold *i*, it was recorded for each person in the study population whether TPC was* bigger* than *i* or not, and the results were analyzed with logistic regression models. In these analyses, two types of logistic regression models were applied: one model type was carried out for each single covariate, with only the given covariate considered as an explanatory variable (the univariate/marginal model) and the other model simultaneously considered all covariates in a multiple logistic regression model (the multivariate/full model). These two models were used in order to uncover whether the covariate was associated with a TPC above* i*, either marginally/in its own respect or in full/considered simultaneously with other covariates, and would implicate that a TPC above *i* would give a different level of disease severity than a TPC below or equal to* i*, within the ICF framework. Significance of all covariates was considered through model reduction. Tests were performed as likelihood ratio tests evaluated with a Chi-square distribution. The statistical significances were tabled as a function of the threshold level and reported as either insignificant or with three varying degrees of significance. In the full model, the *P* values for nonsignificant factors were calculated by adding it to the reduced model and performing the significance test. All logistic regression analyses were carried out using the Splus software, version 6.2 (Insightful Corp. 2003).

## 3. Results

### 3.1. Participants

In the study period, 274 female patients were referred for rehabilitation. Three were excluded due to lack of a sufficient TP examination. This resulted in a study sample of 271 females diagnosed with CWP (1990-ACR definition) and evaluated before enrolment in the rehabilitation program. Due to the large number of variables, it was not possible to obtain complete datasets on all patients. Number of patients with missing values can be extracted from Table 2.

### 3.2. Descriptive Data

**Figure 1:**
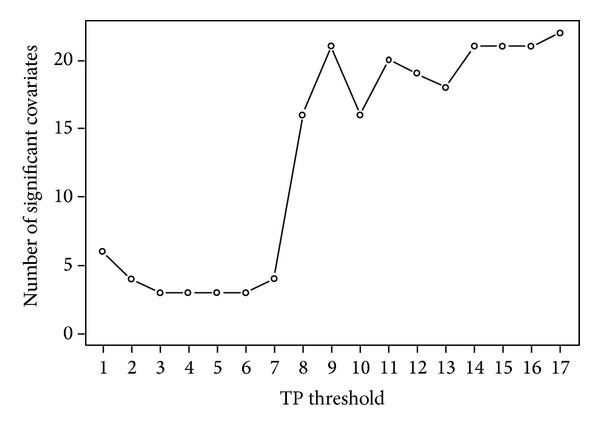
Number of significant covariates versus tender point count (TPC) thresholds, varying between 1 and 17 and with the pain response cutoff set at 2 at the tender point examination. Analyzed in the univariate/marginal model a large number of variables linked to pain and pain-related functional interference (summarized in Table 4) appeared as consistently significant explanatory factors from a TPC threshold at 8, indicating a perturbation of clinical disease severity at this threshold.

Demographic data and key variables classified according to the ICF from the overall study population are presented in Table 2. Setting the TPC pain response cutoff at 1 (mild pain), the median TPC was 18 (range 5–18) and 265 participants (97.8%) had a TPC of ≥11, that is, fulfilling the 1990-ACR criteria for fibromyalgia. Setting the TPC pain response cutoff at 2 (moderate pain), the median TPC was 14 (range 0–18) and 256 participants (94.5%) had a TPC of ≥6 TPs, that is, fulfilling the criteria for fibromyalgia based on the requirement of a moderate or greater pain response at palpation (Figures 1 and 2in Annex A show the number of patients at each TPC with a pain response cutoff set at 1 and 2, resp.).

### 3.3. Threshold Analyses and Multiple Logistic Regression Analyses

The three different multivariate models showed different strength of relationship between variables and number of TPs. In the first model (the continuum model), none of the included variables from the ICF-measurement framework reached a significance level above 0.05 with the TPC established at either of the TPC pain response cutoffs.

Results from the univariate/marginal model, where only the given covariate was considered as explanatory variable, and with the TPC pain response cutoff set at 1 and 2, respectively, are presented in Tables 3 and 4. Setting the pain response cutoff at 1, threshold analysis in the univariate/marginal model revealed only a few scattered significances in the higher end of the TPC threshold spectrum (Table 3). The multivariate/full model did not function for a TPC established at a pain response cutoff at 1. For some thresholds, no interpretable model could be identified, and in the area where marginal significances were present for more than a few covariates (TPC threshold values 14–17), the significances were few and mostly with weak significances (i.e., *P* > 0.01).

With the TPC pain response cutoff set at 2, TPC thresholds ≥8 were associated with a large number of consistently significant covariates related to pain and pain-related interference with everyday life (Figure 1). At TPC thresholds of 1–7 only a few significant covariates were seen and only the* ADL motor ability measure* of* the AMPS *was consistently significant.

A notable feature of the results of the univariate/marginal model with the TPC pain response cutoff set at 2 (Table 4) was that as the TPC threshold increased, all covariates were either consistently nonsignificant, only scattered significant, or reached a threshold from where they became consistently significant.

This was in contrast to the analysis with the TPC pain response cutoff set at 2 in the multivariate/full model (Table 5), where all covariates were considered simultaneously. Due to the high number of variables and a relatively small number of patients with a low TPC, this model could only handle TPC thresholds of 6 and above. For TPC thresholds of 6 and 7, three covariates (*FIQ stiffness, AMPS ADL motor ability, and SF-36 social functioning*) were consistently significant. At the TPC threshold of 8, five new covariates (*total FIQ score, FIQ restorative sleep, FIQ well-being, NSAID use, and BIQ work interference*) entered the model; 4 of them were consistently significant for the next three TPC thresholds when sequentially increasing the threshold value by 1. These covariates, significant for TPC thresholds between 8 and 11, remained significant in the marginal model for higher thresholds, while replaced by other covariates as explanatory variables in the full model. A similar shift was seen for TPC thresholds above 15. These two shifts were comparable to the noted shifts in the marginal model, but where the results in the marginal model were more consistently significant variables, the shift in the full model indicated a change in explanatory variables. Notably, the covariates* social functioning *and* self-reported change or disability from usual working activity* entered the full model as significant explanatory variables at TPC thresholds between 6 and 10, while they were without individual significance in the marginal model.

### 3.4. Comparison between Groups

Based on the results of the regression analyses with the TPC pain response cutoff set at 2, differences between groups were assessed for the TPC threshold of 8 (Table 6).

Within the body domain, patients with a TPC above 8 reported significantly higher levels of pain intensity, tiredness, fatigability, and muscle stiffness and had lower levels of knee muscle strength and walking ability (6-MW) and lower pressure pain thresholds as measured with CPA. However, CPA pain thresholds overall showed only a weak correlation with the number of TPs [pain threshold: *r* = 0.291, *P* = 0.01; pain tolerance: *r* = 0.298, *P* = 0.01]. Measures of psychological distress (anxiety, depression, and pain catastrophizing) were low to moderate in the overall study population and no significant group differences were present.

Within the domain of activity and participation, the TPC threshold of 8 or more was associated with significantly higher self-reported ratings of functional disability and also ADL motor ability measures of the AMPS were significantly lower in patients with a TPC above 8 [1.04 versus 1.29 logits, *P* = 0.004], indicating a higher degree of observable effort and/or fatigability during ADL-task performance. No significant differences between groups were noted for measures relating to the level of participation (e.g., self-reported social functioning or work ability) or personal factors (e.g., ability to reduce or control pain) at this TPC level.

## 4. Discussion

This study provides evidence for a positive relationship between the number of TPs, defined by a moderate or greater pain response to palpation, and disease severity in patients with CWP. Notably, disease expression and TPs at this pain response cutoff were related through a* threshold* on the number of TPs rather than being part of a* continuum*. Logistic regression analyses indicated that a TPC of 8 defined a threshold above which a large number of variables linked to pain and pain-related interference with everyday life became consistently significant explanatory factors (Figure 1).

### 4.1. TPC Threshold Analyses

The manual TP examination represents one method for evaluating the presence and spread of mechanical hyperalgesia [4]. The finding of a positive relationship between a high number of TPs and symptom severity suggests that a widespread distribution of mechanical hyperalgesia influences the expression of clinical symptoms. Notably, we did not find any statistically significant explanatory variables in the continuum model, suggesting that clinical covariates do not impact* continuously* on TPC throughout its range but, rather, in concentrated intervals, in a way that would not be captured by the continuum model. Thus, these results support the notion that disease severity and TPs in patients with CWP are related through a threshold on the number of TPs. A graphic representation of this relationship is seen in Figure 1, which provides a direct visualization that the disease severity does not vary much for TP thresholds less than 8. However, adding another TP to the threshold has a much bigger impact in the interval 8 to 9 TPs compared to the interval 4 to 5 TPs. Therefore, the impact of TP count on disease severity is different between these two intervals, supporting the conclusion that TPC variation should be considered locally (i.e., around a threshold) rather than in a continuum.

Based on the requirement of a moderate or greater pain response at palpation, analyses in the univariate/marginal model showed that a large number of variables from the ICF measurement framework emerged as consistently significant explanatory factors at a TPC threshold ≥8, indicative of a pronounced shift in disease severity at this threshold. The first group of covariates to enter the model was PROs mainly related to pain, fatigue, stiffness, and functioning followed by observation-based measures of muscle strength and walking ability, which appeared at a TPC threshold of 11. The last group of covariates, entering the model at a TPC threshold at 15, were psychological distress variables (anxiety and depression) and self-reported well-being measured with the SF-36. None of these variables appeared as significant explanatory factors at lower TPC thresholds and pain catastrophizing and pain self-efficacy variables never entered the model. This finding was in accordance with the results of the multivariate/full model and supports the notion that the relationship between psychological distress and CWP is not solely due to TPs, but most likely pertains to the affective component of pain.

It is noteworthy that in the univariate/marginal model onlythe ADL motor ability measure of the AMPS was consistently significant throughout the entire range of TPC thresholds. Functional ability is considered a core outcome in clinical pain research [18, 29–31]. A substantial negative impact of CWP on ADL motor ability as measured with the AMPS has been observed in our study population [32]. However, assessment of pain-related interference with functioning is complex and several studies have demonstrated a poor correlation between patient-reported and observation-based assessment of functional ability in patients with chronic pain conditions including CWP [26, 33, 34]. Analyzed in the univariate/marginal model, a significant relationship between TPC and self-reported physical functioning was only demonstrated from a TPC threshold at 8 and above. This finding emphasizes that self-reported and observation-based assessment of functioning may evaluate different aspects of functional ability and the AMPS may prove to be a sensitive and valuable core instrument in the outcome assessment of patients with CWP, particularly at the lower end of the TP spectrum.

TPC threshold analyses in the multivariate/full model with the TPC pain response cutoff set at 2 indicated a shift in explanatory variables similar to the shifts in the univariate/marginal model and at the same thresholds. However, whereas the results in the marginal model were more consistently significant, the behavior of the full model suggested a change in explanatory variables. The results of the multivariate/full model indicated that at lower TPC thresholds covariates related to function (the ADL motor ability measure of the AMPS, muscle stiffness, social functioning, and work interference) seemed to show the strongest and most consistent relationship with number of TPs. Contrary to the findings in the univariate/marginal model, self-reported level of pain and fatigue never entered the full model as significant explanatory factors. Pain reduction following treatment has been shown to parallel improvements in other outcomes in patients with CWP, including self-reported physical and social functioning, sleep, and interference with work [35]. The interaction between TPC, pain, and functional ability is probably multifaceted. Our results indicate that functional ability, whether related to ADL performance or working ability in the multivariate context, provides a stronger correlation to TPC than level of pain. Equivalent to the univariate/marginal model, the next shift in the full model took place for the TPC threshold of 11; the strongest and most consistent relationship now seemed to be with pain threshold measured by CPA. The last shift took place at the TPC threshold of 15; here psychological distress variables including ability to reduce pain, pain tolerance, and self-reported tiredness related to mobility entered the model. The latter has been shown to be an early indicator of later disability and use of social and health services among the elderly [36]. At a TPC threshold ≥11 the full model indicated a strong relationship between pressure pain sensitivity, as measured by CPA and number of TPs. However, there was only a weak correlation between CPA and number of TPs, implying that they probably measure different aspects of mechanical hyperalgesia.

Several studies have reported a positive relationship between number of TPs and self-reported somatosensory symptoms of neuropathic pain in patients with CWP and fibromyalgia [37–39], as well as a positive correlation between neuropathic pain symptoms and pressure pain thresholds measured by CPA [40]. A high TPC or reduced pressure pain threshold, as measured by CPA, points to a predominantly central pain mechanism in CWP pathophysiology. Since somatic and central pain disorders entail different pain management strategies, the identification of augmented pain processing has important implications for therapy. Characterization of patients with CWP, based on the presence and spread of mechanical hyperalgesia, whether assessed by manual TP examination or CPA, may therefore assist in advancing a more individualized and pain mechanism focused treatment in the clinical setting.

### 4.2. Comparison between Groups

Evaluated in an ICF-measurement framework covering core set categories identified for the multidimensional assessment of CWP, the negative impact on measures obtained at the body level and level of activity and participation was substantial. In accordance with the existing literature [41, 42] the results of the study supported a considerable heterogeneity with regard to disease manifestations, as reflected in the observed wide range of scores on key outcome measures. Confirming our hypothesis, patients with a high TPC based on a TPC pain response cutoff set at 2 showed higher levels of clinical pain, muscle stiffness, tiredness, fatigability, and interference with functional ability than patients with a lower TPC. Setting the TPC threshold at 8 yielded significant differences in observation-based tests assessing body functions, including measures of pressure pain thresholds, muscle strength, and walking ability, as well as observation-based assessment of ADL task performance measured with the AMPS. No in-between group differences were present in measures covering the ICF-level of participation, for example, self-reported social functioning or work interference or in psychological distress variables and pain self-efficacy variables at the TPC threshold level of 8, indicating that the pain condition itself and not concomitant psychological distress more likely explained the observed in-between group difference of disease impact.

*Limitations and Generalizability.* The study was conducted in a specialized tertiary-care setting and patients encountered are not necessarily representative of patients from the overall referral population. It has been reported that patients with CWP in referral clinics demonstrate higher pain severity and negative consequences related to pain than similar patient groups found in the community [43]. However, our patient population seems representative of patients encountered in the clinic setting based on the obtained disease severity scores. Reflecting recruitment from a tertiary-care setting only a relatively small number of patients had very few TPs. This influenced the analyses in the multivariate/full model, which could only handle TPC thresholds from 6 and above due to a high number of variables. Finally, the study was limited by only including women. As the prevalence of CWP is higher in women than in men [44] the study results still seem relevant for the CWP population.

## 5. Conclusions

Patients with CWP encountered in the clinical setting exhibit a positive relationship between the number of TPs and severity of the clinical pain condition through a TPC* threshold* rather than a TPC* continuum*. Provided the requirement of a moderate or greater pain response at palpation, the major shift of disease severity occurred at a TPC of ≥8. This relationship was not observed if the TPC in individual patients was based on a TP cutoff set at mild tenderness, suggesting that a hyperalgesic pain response at palpation should be required in order for TPs to be considered a primary identifier of pain hypersensitivity. These findings are at odds with the affective spectrum disorder hypothesis [7] and support the premise that the presence and spread of pressure pain hyperalgesia influences symptomatology in CWP. How to classify or diagnose fibromyalgia is still a matter of debate. Using a TP cutoff at 8, based on the requirement of a moderate or greater pain response at clinical examination, could assist the identification of patients with CWP and a more severe pain condition, which could be labeled as fibromyalgia. Overall, this study supports the continued use of the manual TP examination as a valid measure of pain hypersensitivity and abnormal pain processing in the clinical setting and underlines the need for a comprehensive evaluation in all patients with CWP with a view to early and targeted intervention.

## Supplementary Material

Annex A contains a detailed description of the applied questionnaires and observation-based assessment methods, and two figures showing the distribution of patients according number of tender points with the cutoff for the pain response at tender point examination set at 1 and 2, respectively.

## Figures and Tables

**Table 1:** Instruments classified according to the ICF core sets for CWP.

ICF core sets for CWP	Instruments in this study
Body functions	
Emotional functions	GAD-10, MDI, SF-36, FIQ
Sensation of pain	FIQ, SF-36
Exercise tolerance functions	Mob-T
Psychomotor functions	FIQ, SF-36
Control of voluntary movement functions	AMPS motor
Energy and drive functions	Mob-T, SF-36, FIQ
Sleep functions	FIQ
Content of thoughts	CSQ
Muscle power functions	Grippit, LIDO Multi Joint
Attention function	AMPS process
Activity and participation	
Carrying out daily routines	AMPS, FIQ, SF-36
Handling stress and other psychological demands	CSQ
Family relationships	SF-36
Remunerative employment	FIQ, BIQ
Intimate relationships	
Walking	6-MW
Recreation and leisure	SF-36
Solving problems	AMPS process, CSQ
Lifting and carrying objects	AMPS motor
Doing housework	AMPS, FIQ
Environmental factors	
Drugs	BIQ
Immediate family	BIQ
Health professionals	BIQ
Individual attitudes of immediate family members	
Social security services, systems, and policies	BIQ
Individual attitudes of friends	

GAD-10: generalized anxiety disorder; MDI: major depression inventory; SF-36: short-form-36 health survey; FIQ: fibromyalgia impact questionnaire; CSQ: coping strategy questionnaire; AMPS: assessment of motor and process skills; 6-MW: 6-minute walk; Mob-T: mobility tiredness; BIQ: basic information questionnaire.

**Table 2:** Demographic data and key variables classified according to the ICF model.

	Mean	SD	Range	*N*
Demographics				
Age (years)	45.5	9.66	20.4–71.5	271
Symptom duration (months)	122	102	6–540	227
Body mass index (BMI)	26.9	5.5	16.9–45.7	270

	Never	Sometimes	Daily	*N*

Pain medication				
Weak analgesics	3%	63%	34%	237
NSAID's	39%	43%	18%	230
Opioids	56%	23%	21%	231
	Yes	No		*N*
Antidepressants	31%	69%		237
Pain-related work interference				
Changes or permanent disability from usual working activity due to pain	82%	18%		235
Applied for permanent disability pension at some stage	25%	75%		231
Currently holding a position or enrolled in education	21%			239
Currently on long-term sick leave	25%			239
Currently receiving social benefits or socials services of some sort	34%			239

	Mean	SD	Range	*N*

Body				
Muscle strength UE (PTQ extension)	84.94	36.88	7–199	249
Muscle strength UE (PTQ flexion)	40.23	18.41	5–85	249
Grip strength, max.	174.96	82.24	8–408	258
Vitality (SF-36)	21.88	17.26	0–75	268
Fatigue (FIQ)	8.01	1.95	1.3–10	267
Rest (FIQ)	7.57	2.33	0–10	266
Well-being (SF-36)	55.62	20.61	0–100	268
Well-being (FIQ)	7.32	2.66	0–10	258
Anxiety (FIQ)	4.46	3.43	0–10	267
Anxiety (GAD-10)	19.17	9.78	1–50	268
Depression (FIQ)	3.81	3.42	0–10	268
Depression (MDI)	21.60	10.68	3–50	268
Bodily pain (SF-36)	24.07	15.03	0–84	268
Pain intensity (FIQ)	7.07	1.95	0–10	268
Muscle stiffness (FIQ)	6.25	2.75	0–10	265
Tiredness mobility (Mob-T)	1.68	1.61	0–6	257
Pain detection threshold (PDT)	12.16	7.52	0.3–46.2	229
Pain tolerance threshold (PTT)	31.65	14.73	9.2–86.7	229
Activity and participation				
ADL ability motor (AMPS)	1.07	0.50	0.4–2.82	257
ADL ability process (AMPS)	1.09	0.35	0.12–2.18	257
Walking speed (6-MW)	449.67	114.37	94.6–712.1	259
Activity limitations (FIQ)	5.24	2.22	0–9	267
Physical functioning (SF-36)	42.88	20.01	0–95	268
Role physical (SF 36)	9.71	21.03	0–100	267
Role emotional (SF-36)	45.12	43.30	0–100	263
Social functioning (SF-36)	48.04	26.47	0–100	268
Days of sick leave pr. week (FIQ)	1.12	1.68	0–6	70
Work ability (FIQ)	6.62	2.50	0–10	72
Personal				
Catastrophizing (CSQ)	15.91	8.35	0–36	266
Perceived control over pain (CSQ)	2.40	1.38	0–6	266
Ability to decrease pain (CSQ)	2.28	1.22	0–6	264
Global measures				
FIQ total	61.32	18.53	2.9–97.4	268
SF-36 PCS	26.95	6.68	8.8–50.7	268
SF-36 MCS	40.68	11.96	14.9–66.6	268
General health (SF-36)	31.60	18.15	0–97	267

GAD-10: generalized anxiety disorder; MDI: major depression inventory; SF-36: short-form-36 health survey; FIQ: fibromyalgia impact questionnaire; SF-36 PCS: SF-36 physical composite score; PTQ: peak torque; SF-36 MCS: SF-36 mental composite score; CSQ: coping strategy questionnaire; AMPS: assessment of motor and process skills; 6-MW: 6-minute walk; Mob-T: mobility tiredness.

**Table 3:** Univariate/marginal model: significance of variables at a given tender point threshold with the pain response cutoff set at 1 at the tender point examination.

Outcome variable	TP > 1	TP > 2	TP > 3	TP > 4	TP > 5	TP > 6	TP > 7	TP > 8	TP > 9	TP > 10	TP > 11	TP > 12	TP > 13	TP > 14	TP > 15	TP > 16	TP > 17
Body																	
Muscle strength UE (PTQ ext.)																	∗
Muscle strength UE (PTQ flex.)																∗	∗∗
Grip strength, max.														∗		∗∗	∗∗
Vitality (SF-36)																	∗
Fatigue (FIQ)																∗	
Rest (FIQ)									∗		∗						
Well-being (SF-36)									∗								
Well-being (FIQ)																	
Anxiety (FIQ)																	
Anxiety (GAD-10)																	
Depression (FIQ)																	
Depression (MDI)																	
Bodily pain (SF-36)														∗			
Pain intensity (FIQ)															∗		
Muscle stiffness (FIQ)																	∗
Tiredness mobility (Mob-T)																∗	∗
Pain detection threshold (PDT)									∗	∗					∗	∗∗	∗∗
Pain tolerance threshold (PTT)									∗	∗				∗	∗∗	∗∗∗	∗∗
Activity and participation																	
ADL ability motor (AMPS)														∗	∗		
ADL ability process (AMPS)																	
Walking speed (6-MW)																	∗
Activity limitations (FIQ)														∗	∗	∗	∗
Physical functioning (SF-36)																	
Role physical (SF 36)																	
Role emotional (SF-36)																	
Social functioning (SF-36)																	
Work interference (BIQ)														∗	∗	∗	∗
Personal																	
Catastrophizing (CSQ)																	
Perceived control over pain (CSQ)																	
Ability to decrease pain (CSQ)																	
Analgesics yes/no																	
NSAID																	
Opioids																	
Antidepressants																	
Global measures																	
FIQ total																	
SF-36 PCS														∗			∗
SF-36 MCS																	
General health (SF-36)																	

*P < 0.05; **P < 0.01; ***P < 0.001; GAD-10: generalized anxiety disorder; MDI: major depression inventory; SF-36: short-form-36 health survey; FIQ: fibromyalgia impact questionnaire; SF-36 PCS: SF-36 physical composite score; PTQ flex.: peak torque flexion; SF-36 MCS: SF-36 mental composite score; PTQ ext.: peak torque extension; CSQ: coping strategy questionnaire; PDT: pain detection threshold; AMPS: assessment of motor and process skills; PTT: pain tolerance threshold; 6-MW: 6-minute walk; BIQ: basic information questionnaire.

**Table 4:** Univariate/marginal model: significance of variables at a given tender point threshold with the pain response cutoff set at 2 at the tender point examination.

Outcome variable	TP > 1	TP > 2	TP > 3	TP > 4	TP > 5	TP > 6	TP > 7	TP > 8	TP > 9	TP > 10	TP > 11	TP > 12	TP > 13	TP > 14	TP > 15	TP > 16	TP > 17
Body																	
Muscle strength UE (PTQ extension)								∗	∗		∗	∗	∗	∗∗∗	∗∗∗	∗∗∗	∗∗∗
Muscle strength UE (PTQ flexion)							∗	∗	∗		∗∗	∗∗	∗∗	∗∗∗	∗∗∗	∗∗∗	∗∗∗
Grip strength, max.											∗∗∗	∗		∗∗	∗	∗	∗
Vitality (SF-36)									∗	∗∗	∗∗	∗∗	∗∗	∗∗	∗∗	∗	∗
Fatigue (FIQ)								∗	∗∗	∗∗∗	∗∗∗	∗∗∗	∗∗∗	∗∗∗	∗∗	∗	∗
Rest (FIQ)								∗∗	∗∗	∗∗∗	∗∗∗	∗∗∗	∗∗∗	∗∗∗	∗∗∗	∗∗	∗∗
Well-being (SF-36)															∗	∗	∗
Well-being (FIQ)									∗			∗	∗	∗∗	∗∗	∗	∗∗
Anxiety (FIQ)																	
Anxiety (GAD-10)	∗									∗							∗
Depression (FIQ)																	
Depression (MDI)	∗																∗
Bodily pain (SF-36)				∗				∗	∗∗	∗∗∗	∗∗	∗∗	∗∗	∗∗∗	∗∗∗	∗∗∗	∗∗∗
Pain intensity (FIQ)								∗	∗∗	∗∗∗	∗∗	∗∗	∗∗	∗∗∗	∗∗∗	∗∗	∗∗
Muscle stiffness (FIQ)					∗	∗∗	∗	∗∗	∗∗∗	∗∗∗	∗∗∗	∗∗∗	∗∗	∗∗	∗∗	∗	∗
Tiredness mobility (Mob-T)	∗	∗	∗					∗	∗∗	∗∗	∗∗	∗	∗	∗	∗	∗∗	∗∗
Pain detection threshold (PDT)								∗	∗	∗∗∗	∗∗∗	∗∗∗	∗∗∗	∗∗∗	∗∗∗	∗∗	∗∗
Pain tolerance threshold (PTT)								∗	∗∗	∗∗∗	∗∗∗	∗∗	∗∗∗	∗∗∗	∗∗∗	∗∗∗	∗∗
Activity and participation																	
ADL ability motor (AMPS)		∗	∗∗	∗	∗	∗∗	∗∗	∗∗	∗∗∗	∗∗∗	∗∗∗	∗∗	∗∗	∗∗∗	∗∗∗	∗	∗
ADL ability process (AMPS)						∗					∗						
Walking speed (6-MW)		∗	∗∗	∗∗	∗			∗	∗		∗∗	∗		∗∗	∗∗	∗∗	∗
Activity limitations (FIQ)	∗							∗∗	∗∗∗	∗∗	∗∗∗	∗∗	∗∗∗	∗∗	∗∗	∗∗	∗
Physical functioning (SF-36)								∗	∗∗	∗	∗	∗∗	∗∗∗	∗∗	∗∗	∗∗	∗∗
Role physical (SF 36)																	
Role emotional (SF-36)																	
Social functioning (SF-36)	∗															∗	
Work interference (BIQ)									∗				∗				
Personal																	
Catastrophizing (CSQ)																	
Perceived control over pain (CSQ)																	
Ability to decrease pain (CSQ)		∗															
Analgesics yes/no														∗∗	∗		
NSAID									∗	∗	∗						
Opioids														∗			
Antidepressants							∗		∗								
Global measures																	
FIQ total								∗	∗∗	∗∗	∗∗	∗∗	∗∗	∗∗	∗∗	∗	∗∗
SF-36 PCS								∗	∗∗	∗	∗∗	∗∗	∗∗∗	∗∗∗	∗∗∗	∗∗∗	∗∗∗
SF-36 MCS	∗																
General health (SF-36)																	

*P < 0.05; **P < 0.01; ***P < 0.001; GAD-10: generalized anxiety disorder; MDI: major depression inventory; SF-36: short-form-36 health survey; FIQ: fibromyalgia impact questionnaire; SF-36 PCS: SF-36 physical composite score; PTQ flex.: peak torque flexion; SF-36 MCS: SF-36 mental composite score; PTQ ext.: peak torque extension; CSQ: coping strategy questionnaire; PDT: pain detection threshold; AMPS: assessment of motor and process skills; PTT: pain tolerance threshold; 6-MW: 6-minute walk; BIQ: basic information questionnaire.

**Table 5:** Multivariate/full model: significance of variables at a given tender point threshold with the pain response cutoff set at 2 at the tender point examination.

Outcome variable	TP > 6	TP > 7	TP > 8	TP > 9	TP > 10	TP > 11	TP > 12	TP > 13	TP > 14	TP > 15	TP > 16	TP > 17
Body												
Muscle strength UE (PTQ extension)												
Muscle strength UE (PTQ flexion)							∗		∗∗	∗		∗
Grip strength, max.												
Vitality (SF-36)					∗							
Fatigue (FIQ)												
Rest (FIQ)			∗	∗∗	∗		∗	∗∗∗	∗∗			
Well-being (SF-36)											∗∗	
Well-being (FIQ)			∗	∗∗						∗		
Anxiety (FIQ)									∗			
Anxiety (GAD-10)												
Depression (FIQ)						∗∗	∗	∗			∗∗∗	∗∗∗
Depression (MDI)											∗	∗∗
Bodily pain (SF-36)												∗
Pain intensity (FIQ)												
Muscle stiffness (FIQ)	∗	∗∗	∗	∗∗	∗∗							
Tiredness mobility (Mob-T)											∗∗	∗
Pain detection threshold (PDT)						∗∗	∗∗	∗∗∗	∗∗∗	∗∗		
Pain tolerance threshold (PTT)					∗∗∗						∗∗∗	∗∗∗
Activity and participation												
ADL ability motor (AMPS)	∗∗	∗∗∗	∗∗	∗∗∗	∗∗	∗						
ADL ability process (AMPS)												
Walking speed (6-MW)												
Activity limitations (FIQ)				∗								
Physical functioning (SF-36)				∗								∗∗∗
Role physical (SF 36)					∗							∗∗
Role emotional (SF-36)												∗∗∗
Social functioning (SF-36)	∗∗	∗∗										
Work interference (BIQ)	∗		∗∗	∗∗∗	∗∗							
Personal												
Catastrophizing (CSQ)												
Perceived control over pain (CSQ)												
Ability to decrease pain (CSQ)											∗∗	∗
Analgesics yes/no												
NSAID			∗	∗∗	∗							
Opioids												
Antidepressants											∗	
Global measures												
FIQ total			∗∗∗	∗∗∗	∗∗	∗∗						
SF-36 PCS												∗∗
SF-36 MCS												
General health (SF-36)												

*P < 0.05; **P < 0.01; ***P < 0.001; GAD-10: generalized anxiety disorder; MDI: major depression inventory; SF-36: short-form-36 health survey; FIQ: fibromyalgia impact questionnaire; SF-36 PCS: SF-36 physical composite score; PTQ flex.: peak torque flexion; SF-36 MCS: SF-36 mental composite score; PTQ ext.: peak torque extension; CSQ: coping strategy questionnaire; PDT: pain detection threshold; AMPS: assessment of motor and process skills; PTT: pain tolerance threshold; 6-MW: 6-minute walk; BIQ: basic information questionnaire.

**Table 6:** Variables classified according to the ICF model. Differences between groups at a tender point threshold level at 8 with the pain response cutoff set at 2 at the tender point examination.

Outcome variable	TPC: 0–8		TPC: 9–18		*P* value
	Mean (SD)	Range	Mean (SD)	Range	
Body					
Muscle strength UE (PTQ extension)	96.8 (30.6)	38–160	83.0 (37.5)	7–199	**0.04**
Muscle strength UE (PTQ flexion)	46.6 (17.9)	18–85	39.2 (18.3)	5–84	**0.026**
Grip strength, max.	192.5 (63.61)	76–304	172.2 (84.6)	8–408	0.176
Vitality (SF-36)	26.3 (16.9)	0–70	21.2 (17.2)	0–75	0.105
Fatigue (FIQ)	7.3 (2.2)	1.3–10	8.1 (1.9)	1.4–10	**0.014**
Rest (FIQ)	6.5 (2.5)	0.6–10	7.7 (2.3)	0.1–10	**0.005**
Well-being (SF-36)	55.2 (20.4)	20–92	55.7 (20.7)	0–100	0.904
Well-being (FIQ)	6.6 (2.6)	0–10	7.5 (2.7)	0–10	0.079
Anxiety (FIQ)	3.9 (3.6)	0–10	4.5 (3.4)	0–10	0.328
Anxiety (GAD-10)	17.3 (9.4)	3–42	19.5 (9.8)	1–50	0.217
Depression (FIQ)	4.3 (3.4)	0–10	3.7 (3.4)	0–10	0.348
Depression (MDI)	21.1 (10.7)	5–45	21.7 (10.7)	3–50	0.763
Bodily pain (SF-36)	29.1 (12.3)	0–60	22.3 (15.3)	0–84	**0.034**
Pain intensity (FIQ)	6.4 (1.9)	1.5–9.5	7.2 (1.9)	0–10	**0.021**
Muscle stiffness (FIQ)	4.8 (2.9)	0–9.8	6.5 (2.7)	0–10	**0.001**
Tiredness mobility (Mob-T)	2.4 (1.8)	0–6	1.5 (1.6)	0–6	**0.009**
Pain detection threshold (PDT)	15.0 (8.4)	4.2–46.2	11.7 (7.2)	0.7–42.5	**0.02**
Pain tolerance threshold (PTT)	37.1 (14.2)	12.3–72.8	30.7 (14.6)	9.2–86.7	**0.019**
Activity and participation					
ADL ability motor (AMPS)	1.29 (0.49)	0.11–2.45	1.04 (0.49)	0.04–2.82	**0.004**
ADL ability process (AMPS)	1.16 (0.37)	0.53–1.86	1.08 (0.34)	0.12–2.18	0.204
Walking speed (6-MW)	487.5 (127.3)	142–672	443.8 (111.4)	95–712	**0.035**
Activity limitations (FIQ)	4.2 (2.3)	0–8.7	5.4 (2.2)	0–9	**0.003**
Physical functioning (SF-36)	50.8 (19.3)	10–85	41.7 (19.9)	0–95	**0.012**
Role physical (SF 36)	8.6 (20.14)	0–75	9.9 (21.2)	0–100	0.733
Role emotional (SF-36)	41.2 (41.9)	0–100	45.7 (43.6)	0–100	0.57
Social functioning (SF-36)	52.1 (27.0)	12.5–100	47.4 (26.4)	0–100	0.326
Days of sick leave pr. week (FIQ)	0.89 (1.3)	0–2.9	1.2 (1.7)	0–5.7	0.685
Work ability (FIQ)	5.7 (2.8)	1.4–8.2	6.8 (2.5)	0–10	0.218
Personal					
Catastrophizing (CSQ)	15.3 (8.3)	0–33	16.0 (8.4)	0–36	0.628
Perceived control over pain (CSQ)	2.6 (1.4)	0–5	2.4 (1.4)	0–6	0.354
Ability to decrease pain (CSQ)	2.5 (1.4)	0–5	2.3 (1.2)	0–6	0.284
Global measures					
FIQ total	54.4 (18.59)	14.8–92.1	62.3 (18.4)	2.9–97.4	**0.018**
SF-36 PCS	29.7 (7.0)	15.8–43.8	26.5 (6.6)	8.8–50.7	**0.01**
SF-36 MCS	40.1 (12.6)	20.4–64.3	40.8 (11.9)	14.9–66.6	0.773
General health (SF-36)	34.1 (15.6)	5–77	31.2 (18.5)	0–97	0.4

GAD-10: generalized anxiety disorder; MDI: major depression inventory; SF-36: short-form-36 health survey; FIQ: fibromyalgia impact questionnaire; SF-36 PCS: SF-36 physical composite score; PTQ: peak torque; SF-36 MCS: SF-36 mental composite score; CSQ: coping strategy questionnaire; AMPS: assessment of motor and process skills; Mob-T: mobility tiredness; 6-MW: 6-minute walk.
