# Supplementary material for: The Relationship between Mechanical Hyperalgesia Assessed by Manual Tender Point Examination and Disease Severity in Patients with Chronic Widespread Pain: A Cross-Sectional Study
Source: Int J Rheumatol. 2014 Apr 15;2014:417596. doi: 10.1155/2014/417596 (PMC4009262; doi:10.1155/2014/417596)
Supplement: Supplementary file 1 — Annex A contains a detailed description of the applied questionnaires and observation-based assessment methods, and two figures showing the distribution of patients according number of tender points with the cutoff for the pain response at tender point examination set at 1 and 2, respectively. [file 417596.f1.doc]

**Annex A**


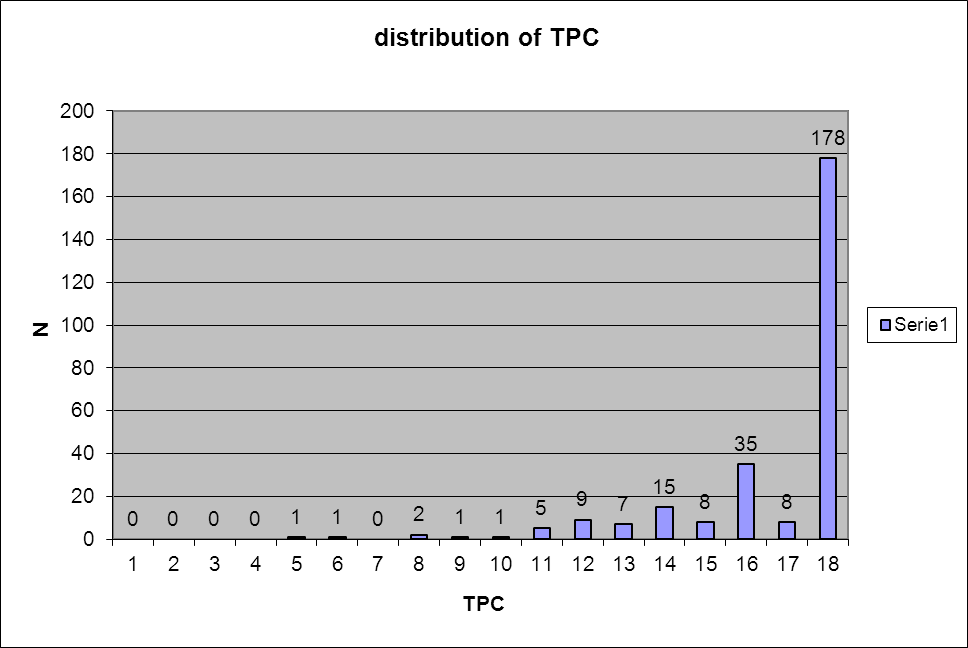


**Figure 1.** Distribution of patients according to number of tender points with the cutoff for the pain response at tender point examination set at 1.

**Figure 2.** Distribution of patients according to number of tender points with the cutoff for the pain response at tender point examination set at 2.

**Instrumentation**

**Patient reported outcomes**

***Short-Form-36 Health Survey (SF-36)***

The SF-36 is a generic, health-related quality of life instrument that assesses eight health domains. All eight domains are scored on a scale of 0-100, with 100 representing the best possible state of health. Two summary scores, the physical component summary (PCS) and the mental component summary (MCS), are provided [12,19].

***Fibromyalgia Impact Questionnaire (FIQ)***

The FIQ is a disease specific, 10-item self-report instrument developed to measure health status in patients with fibromyalgia [5]. The score for each item ranging from 0 -10 can be reported individually or summed to report a FIQ total score ranging from 0-100. A higher score indicates a higher disease impact.

***Generalized Anxiety Disorder (GAD-10)***

GAD-10 is a 10-item self-report inventory developed from the Hamilton 6-item anxiety scale (HAM-A_6_). The instrument measures generalised anxiety according to severity scales by scoring the simple total sum of the items, the total score ranging from 0-50 [3].

***Major Depression Inventory (MDI)***

MDI is a validated 10-item self-report instrument for depression that can be scored both according to the DSM-IV and the ICD-10 algorithms for depressive symptomatology and according to severity scales by the simple total sum of the items, the total score ranging from 0-50 [4]. The instrument has been applied in prevalence studies of major depression in the Danish background population [15].

***The Coping Strategy Questionnaire (CSQ)***

The CSQ is used to evaluate one behavioral and six cognitive coping strategies [18]. Scoring of items on each coping strategy subscale are based on the frequency with which they are used (0=never, 6=always) with a total score ranging from 0 to 36. In addition, there are two self-efficacy items reflecting “perceived control over pain” and “ability to reduce pain” with a score ranging from 0 to 6. Pain studies have found significant relations between both the factor scores and subscales of the CSQ and various measures of adjustment to chronic pain [7]. For this study, only the subscale for Pain Catastrophizing and self-efficacy items were included in the analysis.

***The Mobility-Tiredness (Mob-T) scale***

The Mob-T scale is one of four subscales of the “Measure of functional ability” developed for the elderly population [1]. The Mob-T scale is used to evaluate tiredness related performance of six mobility items. For each item the patients are asked to report if they get tired (0= yes, 1= no) when performing the mobility task. A simple total sum is calculated, the total score ranging from 0 to 6. Low scale values indicate more tiredness related to mobility. Tiredness in mobility has been found to be an early indicator of later disability and use of social and health services among elderly [1,2].

**Observation-based outcomes**

***Maximal isokinetic knee muscle strength***

An isokinetic dynamometer (Lido Multi Joint II, USA) was used to measure maximal voluntary muscle strength of the dominant knee extensors and flexors. Concentric contractions were performed in all patients at an angular velocity of 60°/s and the highest value of 7 repetitions recorded as the maximal muscle strength measured in Nm [11,14,17]. Published norms are available for the Danish background population [6].

***The Grippit® dynamometer***

Grippit® was used to measure maximal grip strength as well as sustained grip strength averaged over a 10 sec period [13]. Grippit® has demonstrated good intra- and inter-rater reliability in healthy adults [8] as well as ability to detect changes in grip strength in patients with fibromyalgia [9].

***Six-Minute Walk Test (6-MW)***

The 6-MW test was standardized and performed in a hospital corridor with a length of 100 meters. Patients were given standard instructions to walk for 6 minutes at a pace that was efficient, but comfortable escorted by a physiotherapist. The distance walked in 6 minutes was recorded in meters. 6-MW testing has been applied in fibromyalgia training studies and found to be reliable in this specific population [10,16].

Reference List

[1] Avlund K. Disability in old age. Longitudinal population-based studies of the disablement process. 2004. University of Copenhagen, Department of social medicine. Institute of publich health.

[2] Avlund K, Damsgaard MT, Sakari-Rantala R, Laukkanen P, Schroll M. Tiredness in daily activities among non-disabled old people as determinant of onset of disability. J Clin Epidemiol 2002;55:965-973.

[3] Bech P, Kastrup M, Rafaelsen OJ. Mini-compendium of rating scales for states of anxiety depression mania schizophrenia with corresponding DSM-III syndromes. Acta Psychiatr Scand 1986;326:1-37.

[4] Bech P, Rasmussen NA, Olsen LR, Noerholm V, Abildgaard W. The sensitivity and specificity of the Major Depression Inventory, using the Present State Examination as the index of diagnostic validity. J Affect Disord 2001;66:159-164.

[5] Burckhardt C, Clark SR, Bennett R. The fibromyalgia impact questionnaire: development and validation. The Journal of Rheumatology 1991;18:728-733.

[6] Danneskiold-Samsoe B, Bartels EM, Bulow PM, Lund H, Stockmarr A, Holm CC, Watjen I, Appleyard M, Bliddal H. Isokinetic and isometric muscle strength in a healthy population with special reference to age and gender. Acta Physiol (Oxf) 2009;197 Suppl 673:1-68.

[7] Hirsh AT, George SZ, Riely III JL, Robinson ME. An evaluation of the measurement of pain catastrophizing by the coping strategies questionnaire. European Journal of Pain 2007;11:75-81.

[8] Lagerström C, Nordgren B. On the reliability and usefulness of methods for grip strength measurement. Scand J Rehabil Med 1998;30:113-119.

[9] Mannerkorpi K, Ahlmén M, Ekdahl C. Six- and 24-month follow-up of pool exercise therapy and education for patients with fibromyalgia. Scand J Rheumatol 2002;31:306-310.

[10] Mannerkorpi K, Svantesson U, Broberg C. Relationships between performance-based tests and patients ratings of activity limitations, self-efficacy, and pain in fibromyalgia. Arch Phys Med Rehabil 2006;87:259-264.

[11] Maquet D, Croisier J, Renard C, Crielaard J. Muscle performance in patients with fibromyalgia. Joint Bone Spine 2002;69:293-299.

[12] McHorney CA, Ware JE, Raczek AE. The MOS 36-Item Short-Form Health Survey (SF-36): II. Psychometric and clinical tests of validity in measuring physical and mental health constructs. Med Care 1993;31:247-263.

[13] Nordenskjöld UM, Grimby G. Grip force in patients with rheumatoid arthritis and fibromyalgia and in healthy subjects. A study with the Grippit instrument. Scand J Rheumatol 1993;22:14-19.

[14] Nørregaard J. Muscle function, psychometric scoring and prognosis in patients with widespread pain and tenderness (fibromyalgia). 1998. University of Copenhagen.

[15] Olsen LR, Mortensen EL, Bech P. Prevalence of major depression and stress indicators in the Danish general population. Acta Psychiatr Scand 2004;109:96-103.

[16] Pankoff BA, Overend TJ, Lucy SD, White KP. Reliability of the six-minute walk test in people with fibromyalgia. Arthritis Care and Research 2000;13:291-295.

[17] Panton LB, Kingsley JD, Toole T, Cress ME, Abboud G, Sirithienthad P, Mathis R, McMillan V. A comparison of physical functional performance and strength in women with fibromyalgia, age- and weight-matched controls, and older women who are healthy. Physical Therapy 2006;86:1479-1488.

[18] Rosenstiel AK, Keefe FJ. The use of coping strategies in chronic low back pain patients: relationship to patient characteristics and current adjustment. Pain 1983;17:33-44.

[19] Ware JE, Sherbourne CD. The MOS 36-item short-form health survey (SF-36). I: Conceptual framework and item selection. Med Care 1992;31 :473-483.
